# Identifying behaviors and factors influencing the prevention of sexually transmitted diseases in young adults

**DOI:** 10.1590/1806-9282.20250634

**Published:** 2025-09-19

**Authors:** Didem Şimşek Küçükkelepçe, Berfin Taşdemir

**Affiliations:** 1 Lokman Hekim University, Faculty of Health Sciences, Department of Midwifery – Ankara, Turkey.; 2 Ankara In Vitro Fertilization Center – Ankara, Turkey.

**Keywords:** Sexually transmitted diseases, Young adults, Health education, Nursing

## Abstract

**OBJECTIVE:**

The aim of this study was to examine the preventive behaviors of young adults regarding sexually transmitted infections and identify the factors influencing these behaviors.

**METHODS:**

This descriptive study was conducted between May and December 2024 with a sample of 385 young women aged 18–25. Data were collected using the Descriptive Information Form and Behavioral Scale for Protection from Sexually Transmitted Diseases. Statistical analysis was performed using SPSS 22.0. Data analysis was conducted using an independent groups t-test, analysis of variance, and posthoc analysis.

**RESULTS:**

The mean total score on the Behavioral Scale for Protection from Sexually Transmitted Diseases was 83.91±9.03 (min: 57; max: 105). Single participants had significantly higher total scores in the information and protection sub-dimension of the Behavioral Scale for Protection from Sexually Transmitted Diseases (p=0.021). Furthermore, those who have received sexual health education demonstrated significantly higher scores (p<0.001). Linear regression analysis indicated that receiving sexual health education accounted for 14% of the total variance.

**CONCLUSION:**

This study highlights the significant role of sexual health education in enhancing awareness and promoting preventive behaviors against sexually transmitted diseases among young adults.

## INTRODUCTION

Sexually transmitted diseases (STDs) are infections caused by pathogens transmitted primarily through sexual contact and are recognized as a major global public health issue^1^. While unprotected sexual intercourse is the primary mode of transmission, STDs can also spread through pregnancy, childbirth, breastfeeding, and exposure to infected blood products^1,2^. According to the World Health Organization (WHO), over one million people worldwide get STDs daily, with many cases remaining asymptomatic. Annually, an estimated 374 million new STD cases occur, and more than 500 million individuals aged 15–49 live with at least one STD^1^. Young adults are considered a high-risk group for STDs due to both biological and behavioral factors^3^. WHO defines adolescents as individuals aged 10–19 years, youth as those aged 20–24 years, and young people as those aged 10–24 years^1,4^. Young adulthood is a critical developmental stage in which individuals explore their sexuality and may engage in riskier behaviors. However, factors such as limited knowledge, fear of stigmatization, misconceptions, and inadequate access to healthcare services contribute to the increased transmission of STDs^5^. Research on STD awareness among young adults suggests that despite educational efforts, their level of knowledge remains insufficient^6^. While students in health sciences generally demonstrate a higher level of knowledge than their peers in other disciplines, studies highlight the need for further educational initiatives^6^. A study on young individuals with substance use disorders found that only 36.8% of participants were aware of STD prevention methods and 61.8% reported not using condoms during sexual intercourse^7^. On the other hand, interventions based on peer education models have been shown to enhance young people's awareness and preventive behaviors regarding STDs^8^. Additionally, sexual health education has been found to positively influence preventive behaviors by providing access to accurate information^9^. In a study, the teaching–feedback method was found to be more effective than face-to-face education in improving STD awareness among women of reproductive age. Various factors at the individual, environmental, and societal levels contribute to increased STD awareness among young adults. Individual-level factors include knowledge, attitudes, and beliefs; environmental influences encompass family, peers, and educational institutions; and social determinants include media, health policies, and cultural norms, all of which directly impact young people's sexual health behaviors^10,11^. To effectively mitigate the spread of STDs, enhancing awareness initiatives, expanding educational programs, and strengthening social support mechanisms for young adults are crucial. This study examined the preventive behaviors of young adults regarding STDs and identified the individual, social, and environmental factors influencing these behaviors.

## METHODS

### Study setting

The study is descriptive and was conducted on online platforms between May and December 2024.

### Population and sample

The study population consisted of young women aged 18–25. The sample size was calculated using the known population sampling method, based on data from the Turkish Statistical Institute (2023), which reported that the population of young people aged 15–25 in Türkiye is 12,949,817. With a 95% confidence interval, a 5% margin of error, and an assumed 50% incidence rate, the required sample size for the study was determined to be 385 women.

#### Inclusion criteria

– Agreeing to participate in the study,
– Being between the ages of 18 and 25.

### Data collection tools and methods

Data were collected using the Descriptive Information Form and the Behavioral Scale for Protection from Sexually Transmitted Diseases (BSP-STD).

### The descriptive information form

It consists of questions about the participants’ age, gender, educational status, current place of residence, socioeconomic level, and information about STDs.

### The behavioral scale for protection from sexually transmitted diseases

The BSP-STD, developed by Kılavuz and Yiğit, consists of 21 items divided into two subscales: knowledge and protection (14 items) and attitude (7 items). It is a 5-point Likert-type scale, with responses ranging from 1 (Strongly Disagree) to 5 (Strongly Agree). The scale ranges from a minimum of 21 to a maximum of 105 points, with higher scores indicating improved positive behaviors toward STD prevention. The Cronbach's alpha coefficient of the original scale was 0.91; in this study, the overall Cronbach's alpha was 0.89.

### Ethical considerations

Ethical approval was obtained from the relevant Ethics Committee (Decision No. 2024/144; Code No: 2024135). Prior to participation, all respondents provided informed consent to take part in the study. This study was performed in line with the principles of the Declaration of Helsinki.

### Data collection

The data were collected online using Google Forms. Participants were recruited voluntarily through the researcher's social media accounts and WhatsApp.

### Statistical analysis

Data analysis was conducted using IBM SPSS 22.0. Descriptive statistics were reported as frequency, percentage, and mean±standard deviation. Group differences were examined using an independent samples t-test, analysis of variance (ANOVA), and posthoc tests. Additionally, linear regression analysis was performed to identify factors influencing protective behaviors against sexually transmitted infections. p<0.05 was considered statistically significant.

## RESULTS

The mean total score on the BSP-STD was 83.91±9.03, indicating a generally high level of positive behaviors toward protection from STDs. The scores ranged from min: 57 to max: 105, demonstrating a broad distribution. Additionally, the mean age of participants was 21.05±2.00 years.

Analysis of the subscales revealed that the mean score for the knowledge and protection sub-dimension was 59.16±6.74 (min: 38, max: 70), suggesting that participants demonstrated a moderate to high level of knowledge and protective behaviors regarding STD. The attitude sub-dimension had a mean score of 24.76±3.24, ranging from 14 to 35 (Table 1).

**Table 1:** Behavioral Scale for Protection from Sexually Transmitted Diseases and age score averages.

	Mean	SD	Min.	Max.
BSP-STD total	83.91	9.03	57.00	105.00
Knowledge and protection subscale	59.15	6.74	38.00	70.00
Attitude subscale	24.75	3.24	14.00	35.00
Age	21.05	2.00	18.00	25.00

BSP-STD: Behavioral Scale for Protection from Sexually Transmitted Diseases; SD: standard deviation.

Total BSP-STD scores based on demographic characteristics revealed no statistically significant differences (p>0.05). However, individuals who had received sexual health education scored significantly higher than those who had not (p=0.000). Regarding marital status, single participants demonstrated significantly higher scores in the knowledge and protection sub-dimension compared to their married counterparts (p=0.021, Table 2). The results of the linear regression analysis showed the BSP-STD score (p=0.000). The explanatory power of the model was assessed, yielding R²=0.140, suggesting that sexual health education accounted for 14% of the total variance in BSP-STD scores. The unstandardized coefficient of the independent variable (B=6.961) indicates that individuals who received sexual health education had an average BSP-STD score 6.96 points higher than those who had not. The standardized coefficient (*β*=0.377) suggests a moderate positive effect of sexual health education on BSP-STD scores. The model's overall significance was confirmed by an F=64,129; p=0.000. Additionally, the Durbin-Watson value (1.899) indicated that the model was within an acceptable range in terms of autocorrelation. The effect of sexual health education remained significant within a 95% confidence interval (5.252–8.670, Table 3).

**Table 2:** Differentiation status of Behavioral Scale for Protection from Sexually Transmitted Diseases scores according to descriptive characteristics.

Demographic characteristics		n	BSP-STH total	Knowledge and protection	Attitude
Education level			Mean±SD	Mean±SD	Mean±SD
High school		77	82.89±8.75	58.45±6.68	24.44±3.20
University degree and above		311	84.16±9.10	59.33±6.75	24.83±3.25
t			-1.103	-1.022	-0.947
p			0.271	0.307	0.344
Marital status					
	Married	34	81.08±8.73	56.61±7.45	24.47±2.37
	Single	354	84.18±9.02	59.40±6.62	24.78±3.31
	t		-1.915	-2.313	-0.535
	p		0.056	0.021	0.486
Employment status					
	Yes	50	81.36±11.11	57.54±8.20	23.82±3.92
	No	338	84.29±8.64	59.39±6.47	24.89±3.11
	t		-2.150	-1.823	-2.194
	p		0.079	0.131	0.069
Income status					
	Income less than expenses	128	83.59±9.85	59.08±7.52	24.50±3.17
	Income equal to expenses	192	83.86±9.15	58.98±6.74	24.88±3.32
	Income more than expenses	68	84.64±6.93	59.77±4.96	24.86±3.16
	F		0.306	0.359	0.554
	p		0.737	0.699	0.575
Receiving sexual health education					
	Yes	154	88.11±8.16	61.70±5.90	26.40±3.16
	No	234	81.15±8.51	57.48±6.74	23.66±2.81
	t		8.008	6.329	8.938
	p		**0.000**	**0.000**	**0.000**
STD history					
	No	376	83.86±8.9	59.12±6.70	24.74±3.24
	Yes	12	85.33±10.50	60.25±7.93	25.08±3.52
	t		-0.553	-0.570	-0.356
	p		0.581	0.569	0.722
Someone diagnosed with an STD in your social circle					
	No	369	83.82±9.08	59.10±6.78	24.72±3.26
	Yes	19	85.57±8.14	60.21±5.96	25.36±2.92
	t		-0.824	-0.698	-0.845
	p		0.410	0.486	0.399

F: Anova Test; t: Independent Groups T-Te. BSP-STD: Behavioral Scale for Protection from Sexually Transmitted Diseases; SD: standard deviation STD: Sexually Transmitted Diseases Bold values indicate statistical significance at p<0.05.

**Table 3:** The effect of receiving sexual health education on Behavioral Scale for Protection from Sexually Transmitted Diseases*.

Independent variable	Unstandardized coefficients		Standardized coefficients	t	P	95%CI	
	B	SE	B			Lower	Upper
Fixed	81.150	0.548		148.186	0.000	80.073	82.226
Receiving sexual health education	6.961	0.869	0.377	8.008	**0.000**	5.252	8.670

* Dependent variable=BSP-STD total score; R=0.377; R^2^=0.140; F=64.129; p=0.000; Durbin-Watson value=1.899 CI: confidence interval; SE: standard error Bold values indicate statistical significance at p<0.05.

## DISCUSSION

The mean age of the participants (21.05±2.00) and the high proportion of university graduates (79.9%) suggest a potential association with better health outcomes and high sexual health awareness, consistent with findings from previous studies^12,13^. Higher education levels play a crucial role in enhancing awareness and knowledge regarding STDs, as supported by prior research^14^. In a study conducted in São Paulo, adolescents with higher awareness levels demonstrated more protective behaviors against HPV and other STDs^15,16^. Public health strategies should emphasize the integration of sexual health education into school curricula and community-based programs. Such programs should aim to enhance awareness, reduce stigma, and improve access to preventive services^15,16^.

However, the high unemployment rate among participants (86.9%) and the fact that 32.9% reported that their income was insufficient to meet their expenses may present significant barriers to accessing healthcare services. Previous studies have demonstrated that limited financial resources restrict access to healthcare, negatively impact health-seeking behaviors, deter individuals from utilizing preventive health services, and contribute to an increased risk of engaging in unsafe sexual behaviors^17,18^.

One of the most notable findings of this study was that 60.7% of participants reported not having received sexual health education. Sexual health education enhances awareness of STDs and promotes preventive behaviors^19^. The present findings indicate that individuals who received sexual health education had higher BSP-STD scores, reinforcing the effectiveness of education in fostering protective behaviors^20^. Furthermore, linear regression analysis revealed that sexual health education accounted for 14% of the variance in BSP-STD scores, underscoring the role of targeted education as a crucial strategy for improving sexual health behaviors among young adults.

Although 96.8% of participants reported never having had an STD, this seemingly low prevalence may be influenced by a lack of awareness or insufficient testing rates. Research indicates that many STDs are asymptomatic, and individuals who do not undergo regular testing may be unaware of their infection status21. Despite this, 79.4% of participants stated that they do not use any method of protection, highlighting a disconnect between knowledge and actual protective behaviors. This inconsistency aligns with findings from previous studies, which suggest that awareness alone does not necessarily translate into preventive action18,20, suggesting that despite possessing knowledge about STDs, the participants do not adequately apply this knowledge in practice. Analysis of BSP-STD scores based on demographic factors indicated that individuals who had received sexual health education and single participants scored higher in the knowledge and protection subscale.

However, no significant differences were observed across other demographic variables. This finding suggests that while demographic factors may not be decisive in influencing STD prevention behaviors, educational interventions play a critical role20,21. Notably, the relatively lower scores in the attitude subscale suggest that although participants had knowledge about STDs and prevention measures, their willingness or motivation to implement these measures might be insufficient22-24. This highlights the need for training programs that not only provide knowledge but also actively work to reshape individuals’ attitudes and perceptions of risk. The findings show the critical role of sexual health education in enhancing awareness and promoting protective behaviors against STDs. Given the participants’ high unemployment rates and financial stress, the results emphasize the need for targeted educational interventions tailored to the specific needs and circumstances of this demographic group. Expanding access to comprehensive sexual health education can help bridge the gap between knowledge and behavior, fostering more positive attitudes and preventive practices. Ultimately, such interventions can contribute to improved health outcomes and a reduction in STD transmission within this vulnerable population.

## CONCLUSION

The study findings underscore the importance of sexual health education in promoting protective behaviors against STDs among young adult women. Interventions targeting awareness and access to health services can significantly contribute to reducing the burden of STDs.

### Limitations

This study has several limitations. First, the data collection was conducted online, which may have limited the representativeness of the sample, as individuals without internet access or interest in sexual health topics may not have participated. Second, the sample consisted only of female participants, which restricts the generalizability of the findings to all young adults. Future studies should include diverse and representative populations to strengthen the external validity.

## Data Availability

The datasets generated and/or analyzed during the current study are available from the corresponding author upon reasonable request.
